# Formative research to optimize pre-eclampsia risk-screening and prevention (PEARLS): study protocol

**DOI:** 10.1186/s12978-025-01980-9

**Published:** 2025-03-24

**Authors:** Nicole Minckas, Alim Swarray-Deen, Sue Fawcus, Rosa Chemwey Ndiema, Annie McDougall, Jennifer Scott, Samuel Antwi Oppong, Ayesha Osman, Alfred Onyango Osoti, Katherine Eddy, Mushi Matjila, George Nyakundi Gwako, Joshua P. Vogel, A. Metin A. Gülmezoglu, Adanna Uloaku Nwameme, Meghan A. Bohren, the PEARLS Trial collaborative group, Nicole Minckas, Alim Swarray-Deen, Sue Fawcus, Rosa Chemwey Ndiema, Annie McDougall, Jennifer Scott, Samuel Antwi Oppong, Ayesha Osman, Alfred Onyango Osoti, Katherine Eddy, Mushi Matjila, George Nyakundi Gwako, Joshua P. Vogel, A. Metin A. Gülmezoglu, Adanna Uloaku Nwameme, Meghan A. Bohren, Amanda Adu-Amankwah, Kwame Adu-Bonsaffoh Kwame, Edna Arends, Kara Blackburn, Lester Chinery, Nathaniel Coleman, Rachel Craik, Alessandra Fleurent, Mark Laws, Robert K. Mahar, Teresiah Njambi Maina, Tetteh Edmund Nartey, Long Nguyen, Jenipher Echenje Okore, Zahida P. Qureshi, Josephine Rabele, Julie A. Simpson, Inge Smit, Ama Asantewaa Tamatey, Alessandra Tomazzini

**Affiliations:** 1 Nossal Institute for Global Health, Melbourne School of Population and Global Health, University of Melbourne, Melbourne, Australia; 2 Department of Obstetrics and Gynaecology, University of Ghana Medical School, College of Health Sciences, Accra, Ghana; 3 Department of Obstetrics and Gynaecology, University of Cape Town, Cape Town, South Africa; 4 Department of Obstetrics and Gynaecology, Faculty of Health Sciences, University of Nairobi, Nairobi, Kenya; 5 Women’s, Children’s and Adolescents’ Health Program, Burnet Institute, Melbourne, Australia; 6 Concept Foundation, Geneva, Switzerland; 7 Department of Social and Behavioural Sciences, School of Public Health, University of Ghana, College of Health Sciences, Accra, Ghana; 8 Department of Obstetrics, Gynecology & Reproductive Biology, Harvard Medical School, Boston, USA; 9 Obstetrics and Gynecology Department, Kenyatta National Hospital, Nairobi, Kenya

**Keywords:** Antenatal care, Artificial Intelligence Ultrasound, Aspirin prophylaxis, Gestational age estimation, Hypertensive disorders of pregnancy, LMICs, Maternal health, Mixed-methods, Pre-eclampsia, Protocol, Risk screening

## Abstract

**Background:**

Pre-eclampsia is a leading cause of maternal and neonatal mortality, affecting nearly 5% of pregnant women worldwide. Accurate and timely risk-screening of pregnant women is essential to start preventive therapies as early as possible, including low-dose aspirin and calcium supplementation. In the formative phase for the “Preventing pre-eclampsia: Evaluating AspiRin Low-dose regimens following risk Screening” (PEARLS) trial, we aim to validate and implement a pre-eclampsia risk-screening algorithm, and validate an artificial intelligence (AI) ultrasound for gestational age estimation. In the trial phase, we will compare different daily aspirin doses (75 mg v 150 mg) for pre-eclampsia prevention and postpartum bleeding. This study protocol outlines the mixed-methods formative phase of PEARLS, which will identify challenges and the feasibility of implementing these activities in participating facilities in Ghana, Kenya, and South Africa.

**Methods:**

We will employ qualitative and quantitative methods to identify factors that may influence trial implementation. In-depth interviews and focus group discussions with policy stakeholders, research midwives, health workers, and pregnant women will explore the barriers, facilitators, and acceptability of pre-eclampsia risk screening, AI ultrasound, and aspirin uptake. A cross-sectional survey of antenatal care and maternity health workers will assess current clinical practices around pre-eclampsia and willingness to participate in the trial activities. Data will be analyzed using thematic analysis and triangulated across sources and participant groups. The findings will inform trial design and help optimize implementation.

**Discussion:**

The research will provide critical insights into the feasibility of pre-eclampsia risk screening and AI ultrasound for gestational age estimation in resource-limited settings. By identifying factors that can influence implementation of pre-eclampsia prevention and care pathways, the findings will inform successful execution of the PEARLS trial, and post-research scale-up activities. This, in turn, can help reduce the prevalence of pre-eclampsia, and improve maternal and newborn outcomes in high-burden settings.

*Trial registration*: PACTR202403785563823 || pactr.samrc.ac.za (Date of registration: 12 March 2024).

## Plain language summary

Pre-eclampsia is a condition in pregnancy that causes high blood pressure and can affect the liver, kidneys, and other organs. It is a major cause of death for women and newborns, especially in low- and middle-income countries. Screening pregnant women early helps identify those at risk so they can start preventative treatments, like low-dose aspirin and calcium.

This is a research protocol for the first phase of our study (called “PEARLS”) which means that it describes what we plan to do and how we plan to do it. PEARLS explores ways to improve pre-eclampsia prevention. PEARLS will: (1) screen pregnant women to identify who is at a high risk for pre-eclampsia, (2) use an artificial intelligence (AI) ultrasound to estimate how many weeks pregnant the woman is, and, (3) compare two doses of daily aspirin (150 mg vs. 75 mg) to see which dose is more effective and safe for preventing pre-eclampsia.

This first phase will take place in Ghana, Kenya, and South Africa. We will interview health workers, policy experts, and pregnant women, and conduct surveys with health workers to understand the challenges of using AI ultrasound, risk screening tools and aspirin for pre-eclampsia prevention. The findings from this phase will help ensure the PEARLS study is successfully carried out in these countries and, ultimately, help reduce pre-eclampsia and improve the health of women and their babies.

## Background

An estimated 4.6% of pregnant women are affected by pre-eclampsia, ranging from 1.0% in the Eastern Mediterranean to 5.6% in African regions [1]. Hypertensive disorders of pregnancy are a leading cause of maternal and neonatal morbidity and mortality, accounting for an estimated 14% of the 287,000 maternal deaths that occur each year globally [2, 3]. A quarter of maternal deaths in Latin America and 10% of maternal deaths in Asia and Africa are due to pre-eclampsia and eclampsia [4].

Pre-eclampsia is a multi-system disorder that develops due to abnormal placentation, dysregulation of angiogenesis, inflammation, oxidative stress, and maternal systemic vascular dysfunction [5–10]. It is diagnosed through identification of new-onset hypertension during pregnancy, in the presence of either proteinuria or new-onset maternal organ dysfunction (thrombocytopenia, elevated serum creatinine or liver transaminases, neurological conditions, or intrauterine growth restriction) at or after 20 weeks’ gestation [6, 8, 9, 11]. There are multiple factors that increase the risk of developing pre-eclampsia, such as history of pre-eclampsia in a previous pregnancy, chronic hypertension, race, advanced maternal age, nulliparity, multiple pregnancy, and co-morbidities such as obesity, diabetes and autoimmune conditions [12–14]. Identifying women who are at increased risk based on the presence of one or more of these factors has formed the historical basis for pre-eclampsia risk screening in antenatal care. Preventive therapies such as low-dose aspirin can be offered to these women identified as high-risk [15].

Available evidence suggests that antenatal pre-eclampsia risk screening and preventive aspirin is not systematically implemented in many low- and middle-income countries (LMICs) [16]. There are multiple reasons for this, including limited access to accurate gestational age assessment, workforce and equipment shortages, and women receiving their first antenatal visit late in pregnancy [17]. Collectively, these challenges mean that many women at risk of pre-eclampsia miss the opportunity to benefit from preventive low-dose aspirin, which should be commenced as early as 11 weeks’ gestation. Furthermore, the optimal aspirin dosage for pre-eclampsia prevention is uncertain—while higher doses (such as 150 mg) might be more effective, the bleeding-related risks of a higher dose have not been quantified. These challenges are explored below, and highlight the urgent need for innovative, context-specific interventions that can improve gestational age assessment, and pre-eclampsia risk screening and prevention.

### Challenge 1: How can we ensure accurate, consistent antenatal risk-screening for pre-eclampsia?

Many guideline and standard-setting institutions—including the World Health Organization (WHO), American College of Obstetricians and Gynecologists (ACOG), and National Institute for Health and Care Excellence (NICE)—recommend routine screening for pre-eclampsia using history-based risk factors only [4, 18, 19]. While history-based screening is relatively easy to implement, it has a low positive predictive value, i.e. many women who are identified as high risk do not go on to develop pre-eclampsia [20–22].

Recently, the Fetal Medicine Foundation (FMF) developed a pre-eclampsia risk screening algorithm that combines history-based risk factors with blood pressure, biomarkers and uterine artery Doppler pulsatility index to estimate pre-eclampsia risk [23, 24]. In a head-to-head study in five European countries, FMF outperformed the NICE history-based criteria, detecting 100% of pre-eclampsia < 32 weeks’ gestation vs 41% at a similar screen-positive rate of 10%, while the ACOG recommendations only detected 6% of preterm pre-eclampsia [21]. The FMF algorithm has also been integrated into a digital clinical decision tool hosted by Tommy’s in the UK. This tool is a web-based, CE-marked medical application to help identify and manage antenatal complications, including pre-eclampsia [25]. Implementation research and scale up of this tool is ongoing in the UK [25] and is planned in other high-income countries.

Although innovations like the FMF algorithm offer solutions for systematic pre-eclampsia risk screening, they remain largely untested in LMICs. Their impact on clinical workflows is unclear—they may improve efficiency, or add complexity to already strained health systems with staff shortages and limited resources [17]. Ethical considerations—including women’s autonomy, their trust in technology, and whether these innovations work in diverse contexts—must also be explored. Tailoring implementation strategies, such as training, optimized referrals, task-shifting, and ethical guidelines, is essential to ensuring health worker and women’s confidence in the tool.

### Challenge 2: How can we improve access to accurate gestational age dating?

WHO recommends that all pregnant women should be offered an antenatal ultrasound scan prior to 24 weeks’ gestation [26]. For many women in LMICs, conventional antenatal ultrasound is unavailable or only offered in selected settings, such as tertiary hospitals or private clinics [27–29]. Barriers include high unit costs for conventional ultrasound systems, inadequate maintenance or repair services, lack of reliable electricity, and a lack of specialist staff who can perform and interpret antenatal ultrasound [27–29]. Artificial intelligence (AI) has enabled new approach to medical imaging, including ultrasound, which can overcome several of these barriers [30–32]. Algorithms trained on high-quality, labelled ultrasound images (where the gold standard gestational age is known) can learn to predict gestational age accurately, without the need for human experts to measure and interpret fetal biometry [32, 33].

Intelligent Ultrasound (IU) has developed an AI-based ultrasound tool—the ScanNav FetalCheck gestational age estimation system [34]. This system aims to provide an accurate point-of-care gestational age estimate, without the need for expert sonology training. Such technologies can also help ensure women at high risk of pre-eclampsia are identified early, and low-dose aspirin commenced.

While AI ultrasound has the potential to reduce some barriers to accurate gestational age estimation in LMICs, its real-world feasibility remains uncertain. Key considerations include the accuracy of AI-generated estimates across population groups (e.g., fetuses of different gestational ages, women from different racial or ethnic backgrounds, women of different body mass indices), the need for health worker training to ensure correct usage and interpretation, and the reliability of the tool in routine clinical practice. For example, antenatal facilities in many LMICs have diverse patient populations, or face limitations in staffing, infrastructure, internet connectivity and electricity. Although research on validity and accuracy of AI ultrasound in antenatal care in LMICs is currently underway, its usability and acceptability need exploration.

### Challenge 3: What is the most effective and safest dose of aspirin for preventing pre-eclampsia?

Prophylaxis with low-dose aspirin is standard treatment for women at high risk of pre-eclampsia [15, 35]. WHO recommends that women at moderate- to high-risk should receive daily low-dose (75 mg) oral aspirin, ideally starting before 20 weeks’ gestation [15]. Aspirin reduces the risk of pre-eclampsia (RR 0.82, 95% CI 0.77–0.88) and fetal or neonatal death (RR 0.85, 95% CI 0.76–0.95) [36]. Despite the compelling evidence of benefit, coverage of preventive aspirin in LMICs remains stubbornly low [37, 38]. A major driver of this is the failure to identify women at high risk of developing pre-eclampsia [39].

Aspirin guidelines internationally range from 75 to 150 mg/day (or 81–162 mg/day in some countries) [40]. Evidence from Cochrane reviews suggests that higher doses (such as 150/162 mg/day) might prevent more pre-eclampsia, yet few dose comparison trials exist [36, 41]. The ASPRE trial by Rolnik et al. [42] used 150 mg/day aspirin compared to placebo, finding a risk reduction of 62% for preterm preeclampsia (RR 0.38; 95% CI 0.20–0.72; p = 0.011) for 150 mg/day of aspirin compared to placebo [42]. However, it remains unclear if the risk of bleeding related to aspirin is greater with a higher dose (such 150 mg/day) as compared to a lower dose (such as 75 mg/day). In terms of safety, a Cochrane review suggests possible increased risk of postpartum hemmorhage  and placental abruption with higher doses, although the confidence intervals touch or cross the line of no effect [36]. Moreover, bleeding-related risks of higher aspirin doses in pregnancy have never been objectively quantified (e.g., using an obstetric blood loss collection drape) [43], and should therefore be interpreted with caution given the known challenges in accurate estimation of postpartum blood loss [44–47]. A 2014 meta-analysis that informed the US Preventive Services Task Force recommendations found aspirin did not affect the risk of postpartum hemorrhage  (RR 1.02, 95% CI 0.96–1.09; seven trials, 22 616 women) [48]. Given these uncertainties, WHO has called for a definitive trial to resolve whether 150 mg is indeed superior to 75 mg, and does not worsen postpartum bleeding [15].

### The PEARLS trial in Ghana, Kenya and South Africa

**Fig. 1:**
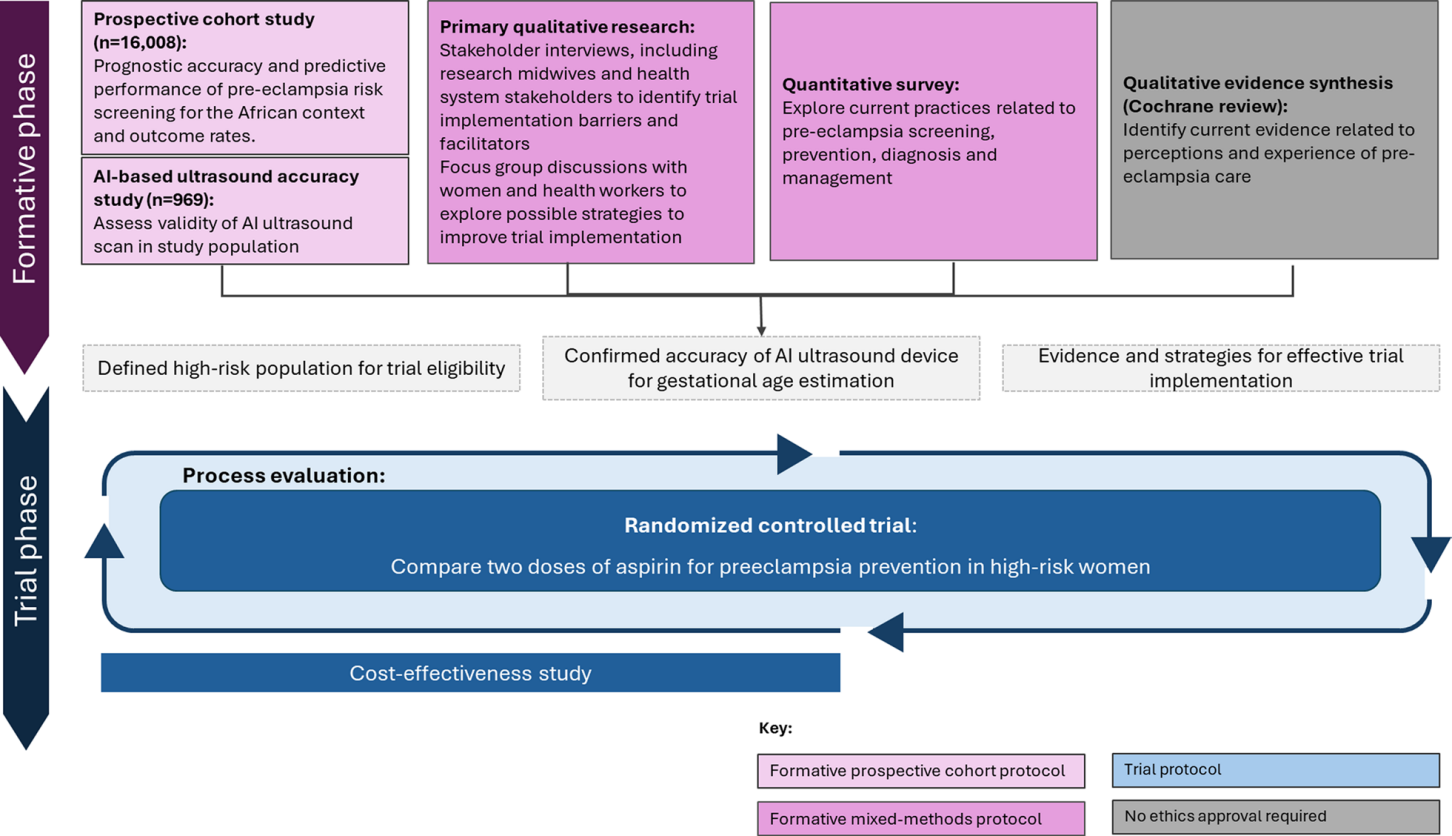
Overview of the PEARLS research activities. The mixed-method components of the project are outlined in this protocol

In order to address these critical challenges to pre-eclampsia risk-screening, gestational age estimation and preventative aspirin dosing, we created the “Preventing pre-eclampsia: Evaluating AspiRin Low-dose regimens following risk Screening” (PEARLS) collaboration, to conduct research with pregnant women and health workers in Ghana, Kenya and South Africa. PEARLS includes (1) a prospective, multi-center, cohort study to evaluate accuracy of pre-eclampsia risk screening, (2) a nested, prospective, multi-center sub-study on accuracy of an AI ultrasound device for gestational age estimation, and (3) an individually randomized, double-blind, comparative effectiveness trial (Fig. 1). The prospective, multi-center, cohort study will estimate the prognostic accuracy and predictive performance of pre-eclampsia risk screening in these settings, using a restricted-variable version of the FMF algorithm. This will be performed using an adapted version of the digital tool used in the UK [25]. The nested, prospective multi-center, sub-study will compare the accuracy of the AI ultrasound to conventional ultrasound for gestational age estimation. Finally, the trial will determine whether 150 mg/day aspirin is more effective and sufficiently safe, compared to the WHO-recommended 75 mg/day dose. These linked studies are essential to informing policy decisions internationally on pre-eclampsia risk screening, antenatal ultrasound implementation and aspirin dosing.

**Fig. 2:**
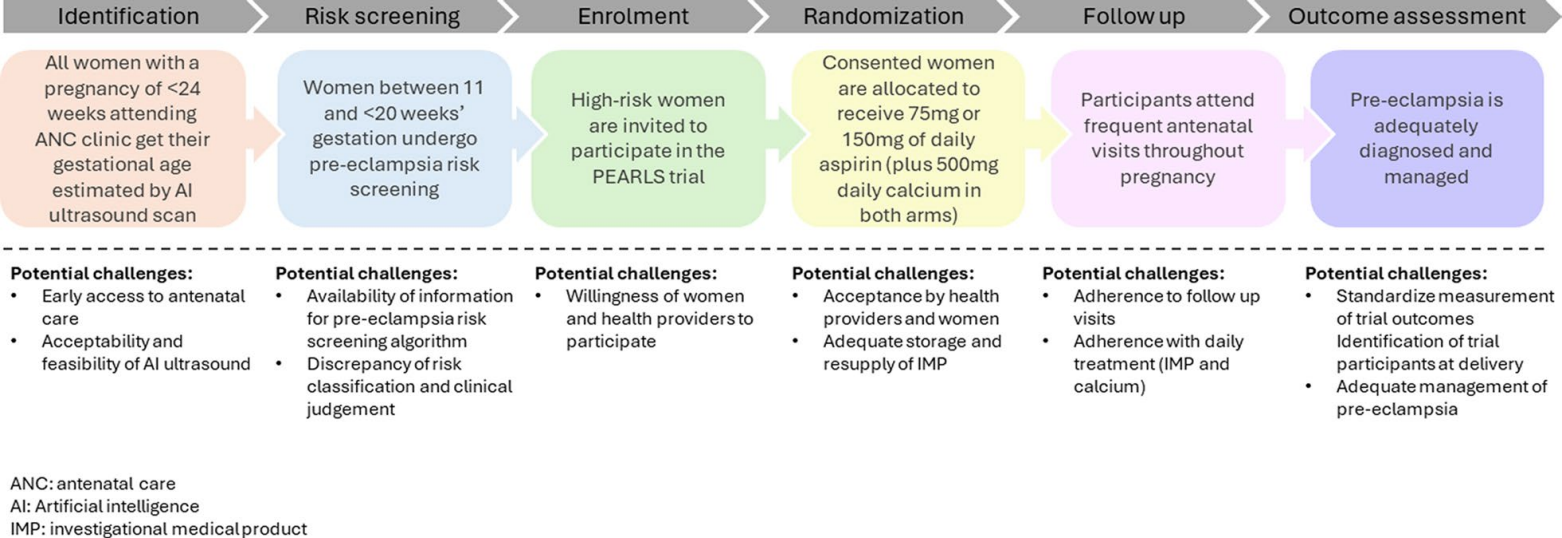
Overview of trial processes and key potential challenges to be explored in the formative phase

Figure 2 is an overview of PEARLS trial processes, highlighting key challenges that may emerge during trial implementation. Given the numerous and complex factors that are likely to affect trial implementation, we designed pre-trial formative research to inform trial design and implementation, and to understand the main factors (e.g., barriers and facilitators) to pre-eclampsia risk screening, AI ultrasound use for gestational age assessment, aspirin use, and diagnosis and monitoring of pre-eclampsia in these settings. Additionally, this research will help develop strategies to improve trial participant recruitment, retention, and follow-up by exploring cultural attitudes towards aspirin use, accessibility of antenatal care, and logistical constraints. This formative study aims to ensure that critical pre-conditions for trial implementation are met before randomization begins and to inform the post-trial of these interventions. Findings will guide future implementation and scale-up strategies after the trial completion, such as community sensitization, mobile health follow-ups, and the inclusion of interventions into existing maternal health services to enhance adherence and minimize participant attrition.

#### Study objectives

Considering the possible challenges to PEARLS trial implementation, the overall aim of the mixed-methods formative study is to optimize pre-eclampsia risk screening and clinical care pathways and identify potential barriers to the implementation of pre-eclampsia prevention strategies, including women’s participation, adherence, retention and follow up. The objectives are:

1. To identify any challenges and optimize implementation of pre-eclampsia risk screening and AI ultrasound in study sites;
2. To understand current clinical practices related to pre-eclampsia screening, prevention and management in study sites; and
3. To assess women’s and health workers’ willingness and possible barriers to participation and retention in the trial.

## Methods

### Study design and sites

This protocol outlines the mixed-methods formative phase (Fig. 2), which aims to inform the implementation of the PEARLS trial and identify potential barriers and facilitators for implementing the post-trial interventions and protocols for the prospective cohorts and trial will be published separately. There are three main activities described in this protocol: (1) qualitative in-depth interviews and focus group discussions (FGD), (2) a quantitative survey, and (3) a qualitative evidence synthesis. The quantitative survey will help establish a baseline of current clinical practices, health worker’s behaviors, and variations across different healthcare settings, offering a broad understanding of current beliefs and practices related to pre-eclampsia. Understanding the barriers, facilitators, and implementation challenges requires qualitative insights, which will explore health worker perspectives, workflow constraints, and acceptability of the interventions. Integrating both approaches will ensure that findings are not only descriptive but also actionable, allowing for the development of tailored strategies to support successful trial implementation and scalability.

All PEARLS activities take place in Ghana, Kenya, and South Africa. Within each country, we selected networks of facilities that provide antenatal and childbirth care. These networks include antenatal care clinics, their linked health facilities where women give birth, and the higher-level hospitals where women with pre-eclampsia are usually referred. Table 1 provides an overview of the number of health facilities within these networks in the three countries. At participating facilities, the trial will provide equipment and hire staff to conduct systematic pre-eclampsia risk screening via tablet, estimate gestational age (informed by the AI ultrasound device), obtain informed consent, and enroll the woman. The mixed-methods formative study will also take place within these same networks of care.

**Table 1:** Networks of care by country

Country	Network of care
Ghana	2 Tertiary referral hospitals 10 District hospitals 4 Primary health facilities
Kenya	5 Tertiary referral hospitals 12 Sub-county hospitals 3 Faith-based sub-county hospitals
South Africa	1 Tertiary Hospital 2 Regional hospitals 1 District hospital 7 Midwife Obstetric Units (MOUs) 4 Basic antenatal care clinics (BANCs)

### Formative qualitative research activities:

#### In-depth interviews with research midwives and key stakeholders

We will conduct stakeholder interviews with two key groups: (1) policy, advocacy, professional society, facility managers and community stakeholders, and (2) research midwives employed at each study site to conduct pre-eclampsia risk screening and use the AI ultrasound device. Interviews will focus on stakeholders’ perspectives on feasibility and acceptability of the risk screening process, strategies to promote early antenatal care attendance, use of aspirin and calcium, additional follow up visits for monitoring, overall diagnosis of pre-eclampsia potential concerns about aspirin use, and engagement strategies. Insights from these discussions will inform practical solutions to enhance participant adherence and minimize loss to follow-up.

In-depth interviews will be conducted with a purposive sample of 10–15 key stakeholders in each country (5–7 research midwives and 5–10 stakeholders per country). At the facility level, research midwives employed in the PEARLS facilities will be invited to participate. We will select them from at least three different facilities, ensuring variation in the size of the facility, location, and other key variables. Other key stakeholders, such as health service managers, advocacy leaders, professional society heads and policymakers will be purposively sampled.

Aligned with qualitative sample size guidelines and principles of thematic data saturation, we will analyze data in parallel with data collection, monitoring for thematic saturation, and adjust the sample size as necessary (e.g. discontinuing further interviews if saturation is deemed achieved, or conducting additional interviews as needed until saturation is deemed achieved). Maximum variation sampling will be used to achieve a stratified sample without random selection and ensure heterogeneity in the participant characteristics. This approach encourages recruitment and sampling based on diversity, specific to the study context.

For research midwives, the country research teams will facilitate contact with potentially eligible participants at their place of work in the study health facilities. For other stakeholders, the country research team will facilitate contact with potentially eligible participants either at their place of work or via email or telephone. Each potential participant will be provided with information about the study, invited to participate by the research team, and if they agree, asked to provide written consent.

#### In-depth interviews procedures and follow-up

Interview guides will be informed by the Capability, Opportunity, Motivation, and Behavior (COM-B) and Theoretical Domains Framework (TDF) frameworks to comprehensively explore and assess all potential factors that may influence behavior change in introducing new pre-eclampsia risk screening processes in clinical practice [49]. The discussion guides will be piloted prior to formal data collection and revisions made to improve clarity. There will be two versions of this discussion guide—one for interviews with research midwives, and the other for key stakeholders.

All stakeholder interviews will be conducted in English via videoconferencing software (e.g. Zoom), telephone or in person, and will take place in a private setting either at the health facility or at the participant's home or workplace. All interviews will be audio-recorded using software or a digital recorder, and the interviewer will take written field notes containing both descriptive information (settings, actions, behaviors) and reflective information (thoughts, ideas, questions, concerns) about the interview. At the start of the interview, participants will be asked to confirm that they have received the information sheet and signed the consent form. Interviews are expected to last approximately 60 min and will be conducted by a trained research midwife or social scientist. Participation in the qualitative study is independent of any subsequent research activities, and responses will not be linked to participants or any identifying information.

#### Focus group discussions

To explore women’s and health workers’ willingness and possible barriers to participating in the PEARLS trial, we will hold FGDs with a sample of women (2–3 FGDs per country) and health workers (2–3 FGDs per country) from selected PEARLS’ facilities in the network of care. For FGDs with women, 6–10 participants will be identified from selected study facilities. The site research team will facilitate contact with potentially eligible participants during their antenatal care visits. For FGDs with health workers, 4–8 participants will be invited from selected PEARLS facilities including midwives, nurses, and doctors working in antenatal care. Separate FGDs will be conducted for nurses/midwives and doctors. Each potential participant will be provided with information about the study, invited to participate by the research team, and, if they agree, asked to provide consent.

#### Focus group discussion’ procedures and follow-up

The FGD guides will be semi-structured, organized around the potential challenges that could come up in the trial and potential strategies to overcome them:

- Potential conflicting processes between conventional care and trial processes.
- Possible barriers and facilitators to early screening, gestational age assessment with AI ultrasound, and overall trial participation.
- Women's communication needs around risk screening.
- Possible barriers and facilitators to the use of low-dose aspirin or calcium.
- Challenges with follow-up monitoring visits and treatment compliance.
- Overall barriers to trial participation, retention, follow up and implementation.

The discussion guides will be piloted before data collection as part of training the research teams, and revisions will be made to improve clarity.

All FGDs will be conducted face-to-face in a private setting in the health facility or other appropriate setting. FGDs with health workers will be conducted in English. FGDs with pregnant women may be conducted in English or in local languages (Swahili, isiXhosa, Afrikaans, Twi). All FGDs will be audio recorded on a digital recorder, and the facilitator will take handwritten field notes containing both descriptive information (settings, actions, behaviors) and reflective information (thoughts, ideas, questions, concerns) about the interview. At the start of the FGDs, participants will be asked to confirm that they have received the study information sheet and signed the consent form. FGDs are expected to last approximately 60–90 min and will be conducted by a trained research midwife or local social scientist. Once the FGDs are conducted, the study participants will not be followed up. Participation in the FGDs will not be contingent on participating in any subsequent research activities, and responses will not be linked by participant or any identifying information.

#### Quantitative survey of health workers

We will invite a sample of antenatal clinic and ward maternity health workers (nurses, midwives, doctors) from the PEARLS facilities to complete an electronic, cross-sectional survey. The survey will cover self-reported current clinical care practices for pre-eclampsia risk screening, prevention, diagnosis and management. We will also seek health workers’ perspectives on potential barriers to risk screening and explore their willingness to support the PEARLS randomized trial.

Potential survey participants are those who currently work in maternity services of study facilities, including (but not limited to) midwives, nurses, junior doctors, medical officers, and obstetricians. Students are not eligible to participate. Potential participants must be capable of reading and responding to the survey questions in English; there are no restrictions on other demographic characteristics of participants. Individuals unable or unwilling to give informed consent to participate will not be able to take part.

PEARLS site teams will generate lists of the total number health workers by cadre and facility. To determine the appropriate sample size for the survey, we will account for the finite population correction, ensuring that the sample is representative of the entire population of health workers in these facilities. The sample size will be calculated using a 95% confidence level, an estimated proportion of 0.5 to ensure maximum variability, and a margin of error of 5%.

The study population will be drawn from three levels of the health system (primary, secondary, and tertiary care) and from different cadres. To ensure that the sample is representative across these levels of care, we will proportionally stratify the sampling based on the actual distribution of human resources in each country’s health system. However, we will intentionally adjust the ratio between obstetricians and midwives/nurses to ensure that all available obstetricians are included in the sample. By including all obstetricians, we aim to capture more comprehensive insights into the diagnostics and management of pre-eclampsia while maintaining a representative sample from the broader health system.

Links to complete the survey will be sent via email to the eligible participant, and reminders to complete the survey will be sent weekly. Participants will also be invited to participate on-site by the research teams. If participants are willing to complete the survey, they will be asked to provide electronic consent via the online survey platform (Qualtrics) before they begin. The electronic consent form has a tick box to say ‘I consent to all of the above statements’ or ‘I do not consent to all of the above statements’. Only those who consent will proceed with the survey. Participants will be asked to complete the survey independently and, in a setting where they feel comfortable giving honest responses without fear of repercussions. The survey is expected to take approximately 30 min to complete.

#### Survey instrument development

The study instrument will be an online quantitative survey using the Qualtrics platform. The survey will be developed by a team of social and behavioral scientists, obstetricians and midwives to ensure clinical relevance and has employed use of behavior change frameworks. The overarching structure and content of the survey will mirror the qualitative interview guide to facilitate triangulation between the key stakeholder interviews and survey responses. The survey will cover the following domains:

1. Sociodemographic information (role, years of experience, country and place of employment);
2. Current practices in pre-eclampsia screening, prevention, and management;
3. Factors influencing current practices for pre-eclampsia screening, prevention and management; and
4. Potential barriers and enablers to implementing digital Clinical Decision tool, the AI ultrasound and use of aspirin.

Response options will include a combination of dichotomous (yes/no or true/false), Likert scales, open-ended short answer response, and multi-option format. The survey will be made available in both a web and mobile friendly format, to enable participants to take part in their own devices with ease. The survey will be piloted prior to data collection, as part of training the research teams.

### Qualitative evidence synthesis

We will conduct a Cochrane qualitative evidence synthesis (systematic review of qualitative research) to describe and explore the perceptions and experiences of women, community members, lay health workers, and skilled health workers who have experience with pre-eclampsia, or with preventing, identifying and managing pre-eclampsia, in both community and health facility settings [50]. This qualitative evidence synthesis will complement the primary qualitative research by including the perspectives of women and communities, and understanding the complexities of pre-eclampsia screening, prevention, diagnosis and management. The Cochrane review protocol will be published elsewhere [50].

### Data management and quality assurance

For the qualitative activities, including interviews and FGDs, all audio recordings and field notes will be stored in a secure, password-protected OneDrive account. Transcription will be carried out either by the data collector or a professional transcription service that complies with General Data Protection Regulation (GDPR) standards. Field notes capturing observations and assessments during interviews or FGDs will be recorded by the interviewer and integrated into the transcripts. Participants will have the option to withdraw their data up to 30 days following the completion of their interview. Should a participant choose to withdraw, their audio recording and written transcript will be permanently deleted and excluded from any future analyses.

For the quantitative survey, data will be collected using Qualtrics, a secure web-based platform that complies with the GDPR 2018. To ensure the confidentiality and integrity of the data, Secure Sockets Layer (SSL) encryption will be employed, creating an encrypted connection between the web server and the user's browser.

### Data analysis

Anonymized transcripts from in-depth interviews and FGDs will be analyzed using a combined inductive thematic and deductive framework analysis [51]. Initially, an inductive thematic analysis will be employed to allow themes to naturally emerge from the data, providing an open exploration of participants' experiences and perceptions [52]. Following this, a deductive framework will be applied, guided by the study objectives and topic guide, to systematically map these emergent themes to predefined key areas relevant to pre-eclampsia risk screening, prevention, diagnosis and management. The identified themes related to implementation factors (e.g., barriers, facilitators, and neutral responses) will be further analyzed using the capability, opportunity, and motivation (COM-B) model and the theoretical domains framework (TDF) [53, 54]. The deductive coding process will involve aligning each theme with the COM-B domain that best represents it. This approach is expected to elucidate key factors influencing the implementation of the digital Clinical Decision Tool and the PEARLS trial, including the design and recruitment strategies.

Quantitative survey data will be analyzed descriptively, utilizing measures such as mean, median, or proportion, and interquartile range (IQR) as appropriate. The results will be presented by key demographic subgroups, including job role, facility level, and country, to facilitate comparative insights. As this study aims to provide an overview of current practices and perceptions rather than test statistical associations, inferential analysis will not be conducted. Instead, this analysis will establish a baseline for current practices and perceptions, enabling comparisons over the course of the PEARLS trial and in the post-trial period [55].

#### Research data triangulation

We will conduct data triangulation to integrate findings from FGDs, stakeholder interviews, surveys, and the qualitative evidence synthesis. This approach will allow us to systematically compare and contrast the data across these sources and participant groups using standardized triangulation methods. The PEARLS research team will tabulate findings from the different data sources, examining areas of agreement, disagreement, and gaps. We will conduct comparisons at three levels: (1) across data sources (FGDs, in-depth interviews, surveys, qualitative evidence synthesis); (2) across cadres (doctors, midwives/nurses); and (3) across countries. The findings from this triangulation exercise will identify barriers and facilitators to trial implementation, which can be targeted by specific implementation strategies or adaptations to the trial operations. To generate these strategies, we will consult tools that integrate the COM-B model and TDF frameworks as part of the Behavior Change Wheel, identifying approaches that are likely to be both relevant and effective in addressing the identified factors [54, 56].

### Ethical considerations

We have received ethics approvals for this study (see Declarations: Ethics approval and consent to participate for list of approvals). The study will employ broad participation criteria to be as inclusive as possible of all cadres of health workers. All potential participants in both the qualitative and survey components will receive information about the study in plain language, conforming to ethical requirements for research involving human subjects. All participants will be free to refuse to participate or stop participating at any time, confidentially, and without prejudice. The contact details of the local investigators, including email address or telephone numbers will be made available to the participants in both the qualitative and survey components, should they require further information and assistance.

Study participants will not receive any compensation for their participation. We expect that the qualitative interviews will take place during their shift at work or from home (if by telephone or Zoom), and they may be provided with light refreshments (such as a cold beverage). Survey participants will not receive any remuneration for their time.

## Discussion

The PEARLS mixed-method formative research aims to inform implementation of a randomized controlled trial on aspirin for pre-eclampsia prevention, which ultimately has the potential to reduce pre-eclampsia prevalence, as well as maternal and perinatal mortality, in high-burden settings. Understanding current practices in the pre-eclampsia care pathway and identifying potential barriers to implementation will also provide critical insight into strategies that could help optimize risk-screening and clinical care pathways, participant recruitment, outcome assessments and treatment compliance. By addressing these objectives, the mixed-methods formative research will inform the conduct of the PEARLS trial, ensuring effective and sustainable implementation across diverse settings, and will provide valuable understanding on potential strategies to improve implementation and scale-up after the trial.

### Expected study outcomes

The mixed-method formative research is expected to yield several key outcomes that will further contribute to the understanding of pre-eclampsia screening and prevention. These include accurate identification of women at high risk of pre-eclampsia, leading to more timely and targeted interventions; the effective use of AI ultrasound for point-of-care gestational age estimation to improve early dating, systematic identification and management of women with high-risk pregnancies; and the identification of barriers and facilitators to aspirin use and pre-eclampsia diagnosis to better understand adherence, follow-up, retention and outcome measurement in the trial phase. These insights will not only inform strategies to overcome trial challenges, but also increases the likelihood of successful trial implementation across study sites. Findings will inform the development of culturally appropriate participant engagement approaches we will use in the trial. The diverse geographical setting of the study ensures that the findings will be widely applicable, providing valuable knowledge into the feasibility and acceptability of implementing these interventions in different contexts. Ultimately, this will enhance the potential for scaling up successful strategies in other LMICs.

### Main problems anticipated and proposed solutions

Health workers and service managers may initially be reluctant to support the implementation of the digital Clinical Decision tool, the AI ultrasound, and the PEARLS aspirin intervention, leading to hesitancy in participating in the formative research on these topics. Health workers might feel uncomfortable expressing concerns, fearing a lack of anonymity. To address this, the study team will emphasize that all responses are anonymized and confidential, reassuring participants that their decision to take part in the mixed-methods formative research will not be shared with peers or line managers. Sensitization activities will also be conducted at each facility to engage top management, securing their support and creating a positive environment for participation. The study team will collaborate with country partners and facility staff to ensure that potential participants who may have reservations are not overlooked.

Additionally, there may be concerns about implementing the interventions within a landscape of shortage of staff, excessive workload and competing studies. To mitigate these issues, the study team will clearly communicate the potential long-term benefits of the interventions, such as improved maternal and newborn outcomes and reduced complications, which could ultimately reduce workloads. During the PEARLS trial, we will conduct a mixed-methods process evaluation to assess the extent to which the trial was implemented as intended (to be prepared as a separate study protocol). This includes assessment of fidelity, acceptability, feasibility, adaptation, and contamination. To support health system integration, study findings from the formative research and process evaluation will be disseminated to policy-makers, regulatory bodies, and professional associations involved in maternal health guidelines and policies. Also, the study team will work with national health authorities in Ghana, Kenya, and South Africa to explore opportunities for embedding risk screening into routine antenatal care and improve resourcing and implementation of aspirin to women screened as high risk. Scalability efforts will consider workforce training, resource allocation, and digital infrastructure needs to ensure long-term sustainability beyond the trial setting.

### Impact on equity and ethics

Improving access to antenatal care, early gestational age dating ultrasound, screening and preventive treatment for pre-eclampsia in high-risk women has the potential to significantly advance health and gender equity, particularly in LMICs where healthcare infrastructure and resources are often limited and maternal morbidity and mortality are highest. The PEARLS trial is designed to address these challenges by implementing and scaling up interventions that expand access to ultrasound, screening, and preventive strategies within health systems that face various barriers. The mixed-methods formative research will play a crucial role in identifying these barriers and developing potential strategies to overcome them, ensuring that interventions are accessible to all cadres of health workers and women, regardless of geographic location or socioeconomic status. By including diverse participant groups from different levels of the health system and various regions, the study seeks to create strategies that are both inclusive and equitable.

Additionally, the integration of AI-driven tools in maternal health raises important ethical considerations. These include women’s ensuring autonomy, the level of trust in algorithm-based diagnostics, and the potential for bias in AI models. The latter may disproportionately impact certain populations if training data do not adequately represent diverse LMIC settings. Ensuring transparency in how AI-derived risk assessments are developed and communicated to both health workers and pregnant women is crucial for fostering informed decision-making and trust in these technologies. Furthermore, it is essential to explore the role of AI in clinical decision-making, balancing automation with health worker expertise to avoid over-reliance on algorithmic outputs. To explore these issues, this study will assess health worker and women’s perceptions of implementing a new risk screening method, identify potential concerns related to decision-making, and explore strategies to enhance the responsible and equitable use of AI in antenatal care. Addressing these ethical and equity-related factors will be fundamental to ensuring the sustainable and culturally-appropriate implementation of AI-based maternal health innovations in these settings. By focusing on inclusivity and equity, the PEARLS trial can help reduce disparities in maternal health, thus supporting the broader goal of improving health equity internationally.

### Private–public and community collaborations

The PEARLS Trial is built on strong collaborations between public and industry partners, bringing together a diverse team of clinicians, multidisciplinary researchers, implementation experts, and women’s health advocates to comprehensively address pre-eclampsia. Industry partners involved in the development of the digital pre-eclampsia risk screening tool and the AI ultrasound system play a crucial role by providing the technological innovations. Their expertise ensures the creation of user-friendly, accurate, and reliable tools, which are essential for the effective implementation of the study’s objectives. Additionally, PEARLS benefits from the collaboration of leading academic institutions, including the University of Ghana Medical School, the University of Nairobi, the University of Cape Town, the University of Melbourne, the Burnet Institute, and Concept Foundation. These institutions contribute rigorous research methodologies and analytical expertise, ensuring that the study is conducted with scientific rigor and is culturally sensitive and acceptable to the target populations. Finally, the PEARLS collaboration is strengthened by engagement with women’s health and pre-eclampsia advocacy groups, including Action on Pre-eclampsia Ghana and WACI Health.

### Next steps

The findings from the mixed-methods formative research will have profound implications for the prevention and management of pre-eclampsia, especially in LMICs. By addressing both technological and behavioral barriers, the study ensures that the interventions are not only feasible but also sustainable and scalable. The next steps will involve disseminating these findings to key stakeholders, including policy-makers, health workers, and community leaders, to inform the development of national and international guidelines for pre-eclampsia prevention and management. Further research will be necessary to explore how these interventions can be integrated into existing healthcare systems, ensuring they are accessible and affordable for all women. This will require close collaboration with governments, non-profit organizations, and other partners to secure the funding and support needed for widespread implementation.

Moreover, gaining a deeper understanding of current clinical practices related to pre-eclampsia screening, prevention, and management will be crucial for informing future guideline updates, protocols, implementation strategies, and scale-up. Ensuring that the perspectives of both pregnant women and health workers are considered will help to make interventions more acceptable and feasible within different community contexts.

In conclusion, the mixed-methods formative research marks a significant advance in the fight against pre-eclampsia. By leveraging the strengths of multiple partners and employing innovative technologies and preventive treatments, the study aims to improve health equity and maternal outcomes worldwide. The insights gained will guide future research and policy development, contributing to the overarching goal of ensuring safe and healthy pregnancies for all women.

## Data Availability

No datasets were generated or analysed during the current study.

## Abbreviations

WHO: World Health Organization
RCOG: Royal College of Obstetricians and Gynaecologists
ACOG: American College of Obstetricians and Gynecologists
IU: Intelligent ultrasound
AI: Artificial intelligence
NICE: National Institute for Health and Care Excellence
LMIC: Low- and Middle-Income Countries
FMF: Fetal Medicine Foundation
RR: Relative risk
CEmONC: Comprehensive emergency obstetric and newborn care
COM-B: Capability, opportunity, motivation, and behavior
FGD: Focus Group Discussion
IDI: In-depth interview
TDF: Theoretical domains framework
GDPR: General data protection regulation
